# Construction of a management protocol for high-risk neurogenic bladder in Chinese patients with type 2 diabetes: a Delphi study

**DOI:** 10.3389/fendo.2025.1603905

**Published:** 2025-10-17

**Authors:** Senying Luo, Wei Ren, YingJie Hu, Ting Deng, Wenjuan Lai, Wenzhi Cai

**Affiliations:** ^1^ Department of Nursing, Shenzhen Hospital, Southern Medical University, Shenzhen, Guangdong, China; ^2^ School of Nursing, Southern Medical University, Guangzhou, Guangdong, China; ^3^ Pulmonary and Critical Care Medicine, Fuyong People's Hospital of Baoan District, Shenzhen, Guangdong, China; ^4^ Department of Urology, Fuyong People’s Hospital of Baoan District, Shenzhen, Guangdong, China; ^5^ Department of Nursing, Peking University Shenzhen Hospital, Shenzhen, Guangdong, China

**Keywords:** neurogenic bladder, type 2 diabetes mellitus, Delphi study, risk stratification, management protocol

## Abstract

**Objectives:**

The objective of this study is to establish an evidence-based protocol for managing high-risk neurogenic bladder (NB) in Chinese patients with T2DM, integrating risk stratification to standardize clinical practice in China region.

**Methods:**

Through a two-round Delphi consensus process involving 20 national experts and evidence synthesis from 13 clinical guidelines and a systematic review, we developed China’s first hierarchical NB risk stratification system. Quantitative analyses incorporated authority weighting (0–1 scale), coordination coefficients, and Kendall’s concordance testing across 81 systematically validated clinical indicators.

**Results:**

High expert engagement persisted through both rounds (Round 1: 90% response rate; Round 2: 94.7%). Consensus levels demonstrated progressive improvement, with primary indicators achieving the most substantial enhancement (Kendall’s W: 0.289 vs. 0.391, 35.3% improvement). Secondary and tertiary indicators showed 5.5% and 27.4% increases respectively (all *p*<0.01). The final protocol reached a consensus, including 4 primary indicators, 17 secondary indicators, and 60 tertiary indicators.

**Conclusion:**

This consensus-driven framework provides innovative clinical tools for NB risk stratification in diabetes care. Its three-tiered structure—integrating policy recommendations, clinical algorithms, and bedside assessment protocols—significantly improves patient management and outcomes, serving as a valuable resource to guide clinical practice.

## 1 Introduction

The rising diabetes pandemic continues to redefine global healthcare priorities. According to the International Diabetes Federation (IDF), an estimated 783 million individuals worldwide will be affected by diabetes by 2045 (1, 2). China accounts for 22% of the global diabetic population, with 118 million cases, of which 96% are type 2 diabetes mellitus (T2DM) (3, 4). This metabolic disorder predisposes patients to multi-organ damage, with complications consuming more than two-thirds of diabetes-related healthcare resources (5). The direct costs associated with diabetes prevention, treatment, and complication management are expected to increase by $337.8 billion by 2030 (6). Currently, the most recent international guidelines for managing type 2 diabetes include the IDF Global Clinical Practice Recommendations for Managing Type 2 Diabetes - 2025 (7)and the American Diabetes Association (ADA) Standards of Care in Diabetes - 2025 (8). In China, the ‘Chinese Guidelines for Diabetes Prevention and Treatment’ (2024 edition) (9)serve as the national standard. However, there is currently no specific expert consensus on the management of diabetic neurogenic bladder (DNB), which consequently hinders the standardized management of this complication.

Among these complications, DNB remains both clinically neglected and highly prevalent, affecting more than 50% of patients with chronic, uncontrolled T2DM (10, 11). Notably, even among patients with well-controlled glycemia (HbA1c ≤7%), 25% still exhibit bladder dysfunction (12). DNB results from progressive nerve damage, leading to bladder dysfunction, and is characterized by four key clinical features, including diminished bladder sensation and impaired detrusor contractility. These features contribute to significant diagnostic delays and complicate treatment (13, 14). The clinical progression of DNB typically occurs in three phases: an initial asymptomatic phase with bladder hypertrophy, an intermediate phase of decompensation with recurrent infections, and an advanced stage of bladder failure requiring surgical intervention (15, 16). If left unmanaged, DNB may lead to severe urinary retention, refractory urinary tract infections (UTIs), and even renal failure, significantly impacting patients’ health and quality of life (17, 18).

Early detection is crucial for preventing complications, yet the clinical implementation of validated predictive models remains suboptimal due to inadequate risk stratification protocols (14). Current methods for predicting the occurrence of DNB include symptom assessment scales (19), predictive models (14, 20), and biomarkers (21). Systematic screening using these models allows for the early identification of high-risk patients, enabling timely intervention to improve long-term health outcomes in individuals with T2DM. Despite advancements in diagnostics, current management strategies face two key challenges: insufficient multidisciplinary collaboration and an over-reliance on late-stage interventions (21, 22).

The Delphi technique is a systematic method for gathering and synthesizing informed opinions from a panel of experts with specialized knowledge in a particular field (23, 24). Recognized as a widely utilized approach for collecting validity evidence, it provides a structured means of engaging expert panels (23).As an alternative to traditional meetings and interviews, the Delphi method facilitates full participation by allowing all participants to contribute equally, thereby ensuring that each individual has an opportunity to influence the decision-making process (25).

This study aims to address two critical gaps in diabetes care: developing a structured prevention framework and integrating predictive models into clinical practice. Using the Delphi method (26), we convened a multidisciplinary expert panel to develop China’s first high-risk DNB management system. Our approach innovatively combines risk prediction with preventive intervention, establishing a four-tiered management protocol: (1) Population-level screening using a validated predictive model; (2) Risk factor management to prevent DNB onset; (3) Intensive bladder rehabilitation for early-stage DNB; and (4) Follow-up and evaluation. This stratified model aligns with emerging precision medicine paradigms while addressing resource disparities within China’s healthcare system.

The clinical implementation potential of this protocol is supported by three key features: (1) Compatibility with existing diabetes management platforms; (2) Stepwise escalation of care intensity based on DNB risk levels; and (3) Integrated quality-of-life metrics for outcome evaluation. By shifting the therapeutic focus from symptomatic management to risk mitigation, this framework could reduce DNB incidence in high-risk populations. Its successful implementation could serve as a model for managing other diabetes-related complications that require early intervention.

## 2 Methods

**Figure 1:**
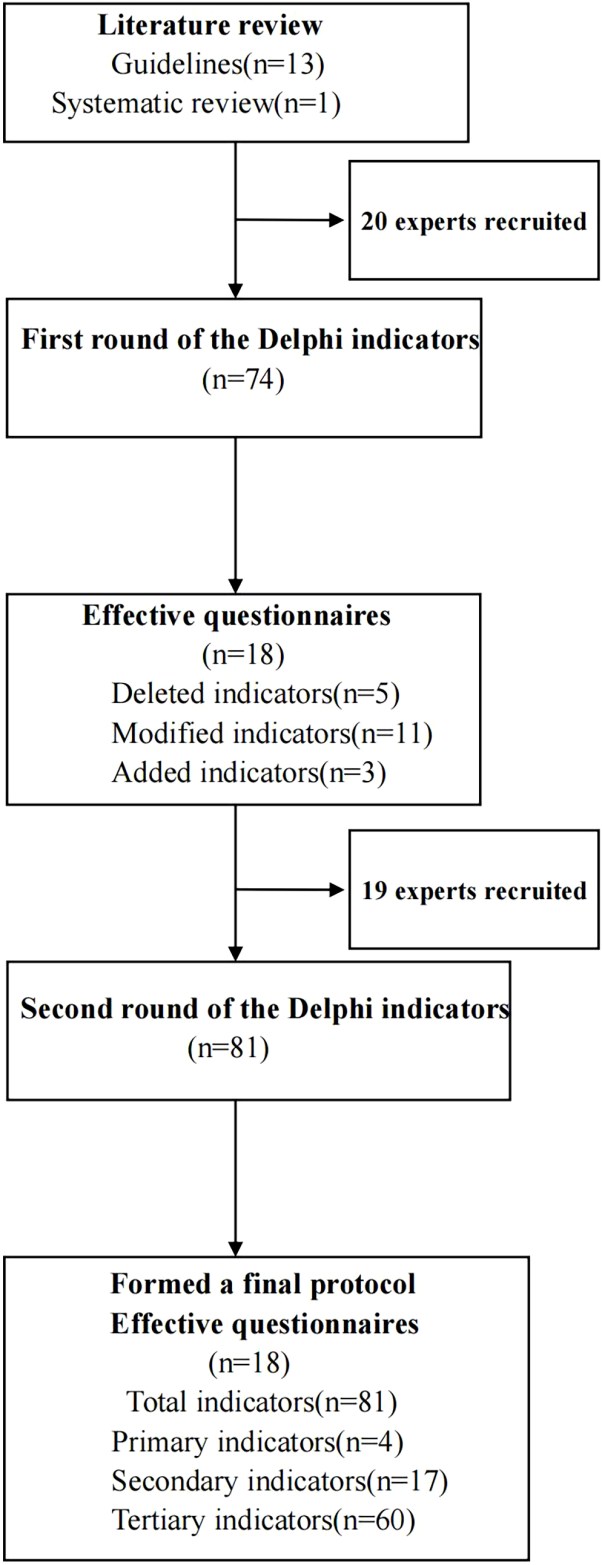
Flow diagram of the Delphi consensus process.

This two-round Delphi study, grounded in a constructivist framework, adhered to the Guidance on Conducting and Reporting Delphi Studies (CREDES) (27) (**Figure 1**). We assembled a research team to conduct literature searches, draft the initial protocol, recruit experts, administer advisory questionnaires, analyze feedback from experts in both rounds, and make necessary revisions to the protocol.

### 2.1 Research team establishment

The research team comprised 11 multidisciplinary members: an endocrinologist, a urologist, a rehabilitation physician, four clinical nurses (two from rehabilitation medicine, one from endocrinology, and one from urology), a statistician, and three graduate students. Among the seven clinical staff members, four held intermediate professional titles, while three possessed senior professional titles. The team was primarily responsible for discussing and defining evaluation indicators, preparing expert consultation questionnaires, and systematically organizing, analyzing, and critically evaluating expert feedback.

### 2.2 Literature search

The team adhered to the “6S model,” systematically searching from top to bottom across its hierarchical layers to comprehensively retrieve relevant evidence. The databases searched included BMJ Best Practice, UpToDate, the Joanna Briggs Institute (JBI), the National Institute for Health and Clinical Excellence (NICE), the Scottish Intercollegiate Guidelines Network (SIGN), the Cochrane Library, and PubMed. Additionally, Chinese databases such as the Yimaitong Guideline Network, China National Knowledge Infrastructure (CNKI), Wanfang, Weipu, and the Chinese Biomedical Literature Database (CBM) were utilized. The search covered publications from the establishment of these databases up to June 30, 2024, with a language restriction to Chinese and English. The search strategy combined MeSH descriptors with unrestricted search terms. The English search terms employed were: (diabetes OR diabetic) AND (neurogenic bladder OR bladder dysfunction OR lower urinary tract symptoms OR lower urinary tract dysfunction OR cystopathy OR overactive bladder OR urinary urgency OR urinary frequency OR urinary incontinence OR urinary retention) AND (prevention OR intervention OR care OR management OR risk).

The inclusion criteria for the literature were as follows: (1) the study population consisted of patients with T2DM aged ≥18 years; (2) the literature addressed risk assessment, prevention, screening, management, or intervention strategies related to DNB; and (3) the outcome measures included the incidence of DNB and improvements in bladder function. The exclusion criteria were: (1) studies involving patients with concurrent conditions such as benign prostatic hyperplasia, female urinary incontinence, spinal cord injury, multiple sclerosis, or bladder cancer; (2) literature types categorized as study protocols, reports, abstracts, case descriptions, reviews, or cross-sectional studies; (3) studies employing pharmacological or surgical intervention methods; and (4) research that did not adhere to quality assessment standards.

**Figure 2:**
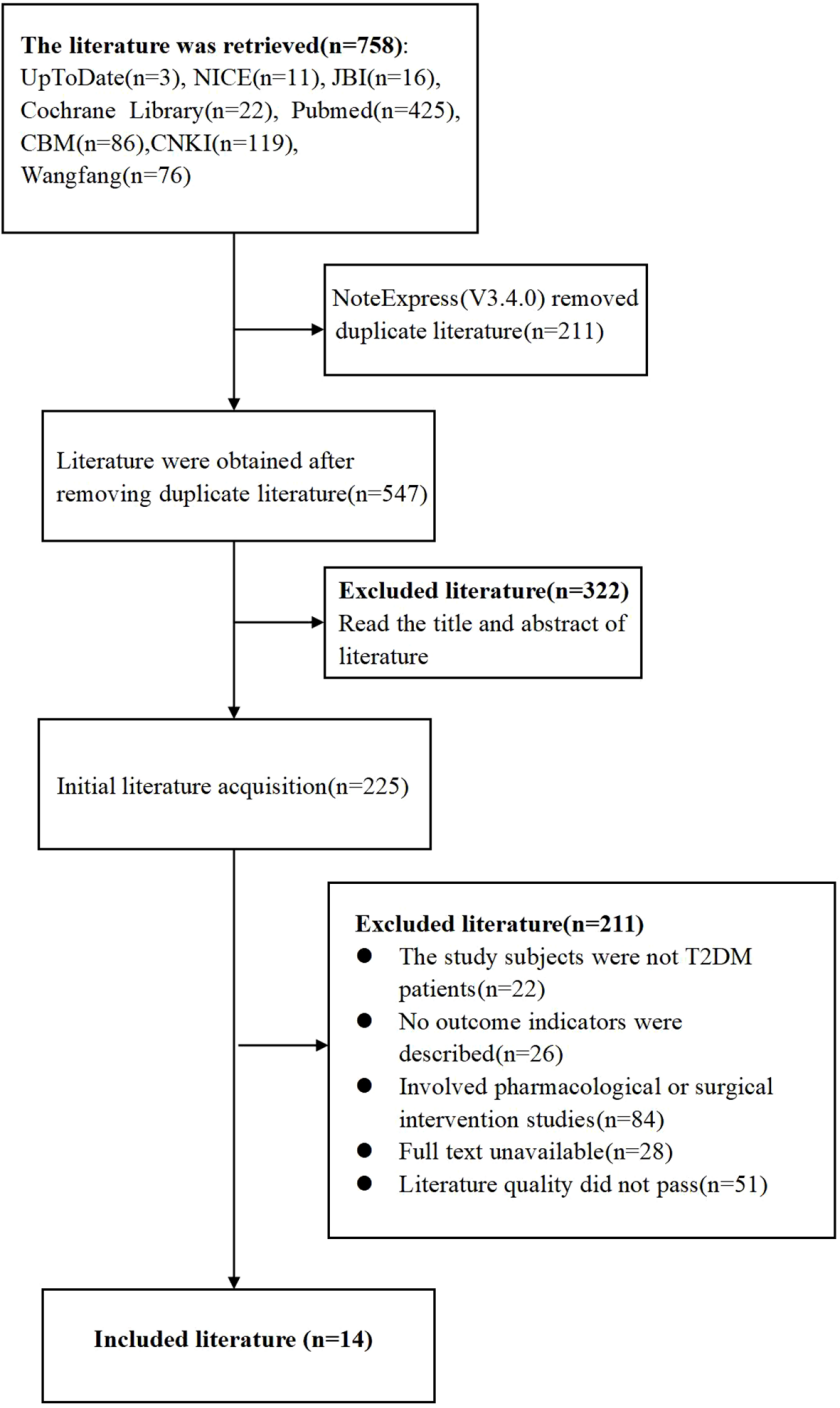
Flow chart of literature screening.

This study retrieved a total of 758 literature items, comprising 281 articles in Chinese and 477 articles in English. After importing into NoteExpress (V3.4.0) and removal of duplicates, 547 articles remained. Two team members independently read the title and abstract of each article. After this step, 225 articles remained. Further reading of the full text and removal of 211 articles that did not meet the inclusion criteria of this study resulted in 14 articles included in the final analysis (**Figure 2**). Following this selection process, 14 studies were included, comprising 13 guidelines and one systematic review.

### 2.3 Development of the protocol

#### 2.3.1 Initial draft

Building on the DNB risk factors identified in prior research (14) conducted by the study team and the literature review, four dimensions of DNB risk management were defined: clinical assessment, risk management, health behavior intervention, and follow-up and evaluation. Subsequently, the research team developed a protocol based on evidence synthesis, literature reviews, and group discussions, incorporating the DNB risk prediction model established in previous studies (14). Finally, the initial management protocol comprised 4 primary indicators, 15 secondary indicators, and 55 tertiary indicators.

#### 2.3.2 Advisory questionnaire

The study questionnaire comprised four primary components: (1) an introduction outlining the background and objectives of the study; (2) basic demographic and professional information about the experts, including age, gender, position, educational background, academic qualifications, specialty, and years of clinical experience; (3) the main questionnaire, which presented a list of indicators with corresponding scores and required experts to rate the importance of each item using a 5-point Likert scale (ranging from “very important” to “not important”). A blank section and a comment box were included to allow experts to suggest modifications based on the relevance and importance of the indicators; (4) an assessment of the experts’ familiarity with the field and the rationale for their judgments. The expert consultation questionnaires were developed using Wenjuanxing (www.wjx.cn) (28), a web-based survey platform, and distributed via email. To ensure technical reliability, the responsive design template underwent rigorous validation across various devices (desktop/smartphone) and browsers (Chrome/WeChat) before distribution. Additionally, a PDF backup mirroring the digital format was provided to mitigate connectivity barriers.

#### 2.3.3 The panel of experts

The inclusion criteria for experts were as follows: (1) extensive practical experience in the field of DNB, with over 10 years of experience in relevant departments such as rehabilitation, urology, endocrinology, and health management; (2) possession of a bachelor’s degree or higher; (3) holding an associate senior or higher professional qualification; and (4) willingness to participate in this project.

#### 2.3.4 Implementation

##### 2.3.4.1 First round

The research team contacted the experts and, upon obtaining their consent, distributed the consultation questionnaire via email, allowing a 7-day response period. If experts did not respond within this period, a reminder email was sent to encourage their participation. Experts were instructed to complete the questionnaire according to the provided guidelines, with the option to modify the content based on their experience and knowledge, as well as to offer additional suggestions. After collecting the completed questionnaires, statistical analysis was performed to develop the second-round questionnaire.

##### 2.3.4.2 Second round

The second round of consultation was conducted four weeks after the first round (29). In this round, the indicators in the main questionnaire were comprehensively analyzed and summarized based on the results of the previous round. The criteria for selecting indicators included an importance score greater than 4.0 and a coefficient of variation less than 0.25 (30)). Indicators that did not meet these criteria were either revised or removed following consultation with advisory experts or through internal discussions. In instance where indicators were disputed, an authority-weighted consensus approach was used to arrive at resolution (31). After revising the primary consultation questionnaire, the second round of the consultation questionnaire, along with an analysis of the first round results (importance scores, frequency of maximum scores, and coefficient of variation), participants’ responses, and all received comments, were sent to the experts via email (32). Seven days were designated for collecting responses; if experts did not respond within this period, a reminder email was sent to them. In total, two rounds of Delphi consultation were conducted, and consensus was achieved when experts expressed similar views on the indicators, demonstrating acceptable consistency.

#### 2.3.5 Weight assignment of indicators

We calculated the weights using a systematic approach. Initially, expert scoring data for the indicators were organized and entered into SPSSAU software to create the precedence chart weight tables (33). The Precedence Chart Method was then applied to derive the weights for the primary, secondary, and tertiary indicators. Finally, the Continuous Multiplication Method was used to compute the combined weights of the secondary and tertiary indicators.

#### 2.3.6 Data analysis

Data entry and analysis were performed using Excel 2019 (Microsoft Corp.) and SPSS 26.0 (IBM Corp.), respectively. Response rates were calculated to evaluate the experts’ positive attitudes toward the research, defined as the ratio of returned questionnaires to distributed questionnaires. A response rate exceeding 70% was considered indicative of effective consultation (34). Expert authority on the topic was assessed by calculating the composite reliability (Cr), derived from the mean values of self-evaluated familiarity (Cs) and judgment basis (Ca) coefficients. A composite reliability (Cr) value ≥0.7 was deemed to indicate reliable expert authority (35). Consistency was evaluated using the coefficient of variation (CV) and Kendall’s coefficient of concordance (*W*) (11, 36, 37). Retention criteria for indicators included a CV <0.25, a mean importance score >4.00, and a full score ratio >20% (38). Indicators failing to meet these thresholds were modified or excluded based on feedback from consulting experts or internal discussions.

#### 2.3.7 Ethical approval

The study received approval from the Clinical Research Ethics Committee of Fuyong People’s Hospital in Shenzhen (KY-2024-10) and was conducted following the Declaration of Helsinki. Nevertheless, before participation, individuals were informed about the study’s purpose, content, and methodologies, and they provided their consent to partake in the research. Participants were assured that they could withdraw from the study at any time without any repercussions, and their data were anonymized to ensure confidentiality, being utilized solely for statistical analysis purposes (36, 39).The consent process was documented in the study records by the research team, including the date and a unique participant identifier.

#### 2.3.8 Quality control

Two members of the research team reviewed the returned questionnaires. Any questionnaire with a response rate lower than 92.5% was excluded (36). Experts were unable to access the consultation results from other experts (40). Once all consultation questionnaires were returned, the research team discussed each comment based on subjective judgment and a literature review.

## 3 Results

### 3.1 Expert panel composition

A multidisciplinary panel of 20 nationally recognized experts was systematically recruited from seven tertiary referral centers across four major cities in China’s Greater Bay Area—Guangzhou, Shenzhen, Jiangmen, and Hong Kong. This region was prioritized for several reasons: (1) the availability of advanced medical resources that are among the best in China, (2) its pioneering role in developing multidisciplinary diabetes care models, and (3) the representation of diverse socioeconomic and healthcare delivery systems. The panel comprised seven essential disciplines: endocrinology physicians, diabetes specialist nurses, urology physicians, urology specialist nurses, rehabilitation therapists, rehabilitation specialist nurses, and health management specialists. The panel represented four institutional types: university teaching hospitals (n=3), public tertiary hospitals (n=2), private non-profit hospitals (n=1), and specialized rehabilitation centers (n=1).

Round 1 dynamics

Of the 20 initial invitees, one non-respondent withdrew due to scheduling conflicts, and another was excluded due to incomplete data (<92.5% item completion) (36), in accordance with pre-specified quality control criteria. Therefore, a total of 18 valid questionnaires were returned, resulting in a 90% valid response rate. The panel consisted of experts from the following fields: Endocrinology (n=4), Urology (n=5), Rehabilitation medicine (n=8), and Health management (n=1). Participants demonstrated substantial field experience (mean ± SD: 24.1 ± 8.8 years) and academic qualifications, with 50% holding senior professional titles.

Round 2 engagement

All 19 eligible experts (one exclusion due to first-round non-response) were re-invited, with 18 completing evaluations, resulting in 94.7% retention. Notably, the initially excluded expert actively contributed valid input during this phase. This round saw a shift in gender composition, with the proportion of female participants decreasing from 66.67% to 61.11%. This change is attributed to the absence of one female rehabilitation medicine expert, while the previously excluded male urology expert rejoined and provided valuable input. The iterative process maintained high engagement levels, with the final analysis including 18 complete paired responses. The expert characteristics are detailed in **Table 1**.

**Table 1:** Characteristics of experts.

Variable	First round		Second round	
	N	%	N	%
Age (years)				
30–39	4	22.22	4	22.22
40–49	9	50.00	9	50.00
≥50	5	27.78	5	27.78
Gender				
male	6	33.33	7	38.89
female	12	66.67	11	61.11
Educational attainment				
Bachelor’s degree	10	55.56	9	50.00
Master’s degree	6	33.33	7	38.89
Doctorate	2	11.11	2	11.11
Years of clinical experience				
10-19	7	38.89	7	38.89
20-29	6	33.33	6	33.33
≥30	5	27.78	5	27.78
Professional qualifications				
Associate Senior	9	50	9	50
Senior	9	50	9	50
Professional area of expertise				
Endocrinology	4	22.22	4	22.22
Urology	5	27.78	6	33.33
Rehabilitation medicine	8	44.44	7	38.89
Health Management	1	5.56	1	5.56

### 3.2 Coefficient of authority, consistency, and coordination degree of experts

In the initial round of consultation, expert reliability was high, with a coefficient of authority (Cr) of 0.894, indicating strong consensus. The coefficient of variation (CV) for these indicators ranged from 0.048 to 0.236, indicating variability in the responses. The Kendall’s coefficient of concordance (*W*) for the primary, secondary, and tertiary indicators was 0.289 (*p <*0.01), 0.200 (*p*<0.001), and 0.175 (*p*<0.001), respectively. These results indicate a weak agreement across the indicators (41, 42). Furthermore, the statistical significance of these *W* values highlights the varying degrees of agreement among the experts.

In the subsequent round of consultation, the values for self-evaluated familiarity (Cs), judgment basis (Ca), and Cr were updated to 0.878, 0.950, and 0.914, respectively (**Table 2**). The CV for the indicators in this round ranged from 0 to 0.212, suggesting a change in the consistency of the responses. The Kendall’s *W* values for the primary, secondary, and tertiary indicators in this round were recorded as 0.391(*p*<0.001), 0.211(*p <*0.001), and 0.223(*p*<0.001), respectively. The improvement in the Kendall’s *W* value for primary indicators from 0.289 in Round 1 to 0.391 in Round 2 indicates a significant enhancement in consensus among experts. However, it is important to note that with a *W* value of 0.211 and 0.223 for secondary and tertiary indicators, respectively, the level of expert agreement remains classified as weak (41, 42). Despite this weak consensus, the findings are statistically significant. These results are detailed in **Table 3**.

**Table 2:** Degree of authority of the experts.

Round	Judgment basis (Ca)	Self-evaluated familiarity (Cs)	Coefficient of authority (Cr)
Round 1	0.911	0.878	0.894
Round 2	0.950	0.878	0.914

**Table 3:** Consistency and coordination degree of expert opinions.

First round				Second round		
Indicators	Kendall’s W	χ²	*P*	Kendall’s *W*	χ²	*P*
Primary indicators	0.289	15.612	<0.01	0.391	21.092	<0.001
Secondary indicators	0.200	50.324	<0.001	0.211	60.764	<0.001
Tertiary indicators	0.175	169.765	<0.001	0.223	236.295	<0.001

### 3.3 Indicator refinement

In the first round, 12 experts provided feedback, resulting in a total of 18 proposed revisions to the indicators. Although all indicators met the predefined quantitative consensus thresholds in Round 1 (mean importance score >4.0; CV <0.25 and full score ratio >20%), qualitative expert feedback identified opportunities to enhance operational clarity and clinical applicability (32, 43, 44). Based on this feedback and subsequent group discussions, several items were revised.

#### 3.3.1 Primary indicators

Experts suggested changing “assessment” to “clinical assessment” and “health behavior change intervention” to “health behavior intervention,” as these terms are more precise and their semantics clearer.

#### 3.3.2 Secondary indicators

Experts suggested revising “renal function abnormalities” to “diabetic kidney disease (DKD)” to align with the KDIGO diagnostic criteria (45). In the management of risk factors, some experts suggested including “high BMI,” “urinary tract infections,” and “diabetic retinopathy.” Additionally, it was recommended to consolidate “diabetic nephropathy,” “diabetic retinopathy,” and “diabetic neuropathy” into a single category termed “diabetes-related microvascular complications.” Furthermore, some experts proposed dividing “follow-up content and frequency” into two distinct indicators: “general follow-up content and frequency” and “specialist follow-up content and frequency.”

#### 3.3.3 Tertiary indicators

In the tertiary indicators, some experts highlighted that the description of “1.1.1 Endocrinologists evaluate the patient’s medical history, physical examination, auxiliary examination findings, and medication use” was vague and lacked practical applicability, recommending further refinement. Additionally, the experts proposed incorporating “complication screening” into “1.1 General assessment.” For “1.2 Specialist assessment,” it was suggested to include evaluations of “urinary system management” and physical examinations of the “urogenital system and nervous system.”

Regarding “2.1 Hyperglycemia,” one expert recommended introducing “Time in Range (TIR) for glucose” as a control target. Another expert suggested removing “2.1.1 Management of hyperglycemia includes five key measures: medical nutrition therapy, exercise therapy, blood glucose monitoring, diabetes education, and the use of hypoglycemic agents, following the strategies outlined in the 2020 Edition of the Guidelines for the Prevention and Treatment of Type 2 Diabetes in China” and “2.1.3 Initiating medication therapy when blood glucose cannot be controlled through lifestyle interventions alone,” citing redundancy with the content in “3.2 Lifestyle intervention.” Similarly, the experts recommended removing the “lifestyle intervention” sections in “2.2 Hypertension” and “2.3 Hyperlipidemia” due to content overlap.

The experts recommended removing “2.2.4 Antihypertensive drug treatment” and “2.3.3 Lipid-lowering drug treatment,” as these responsibilities primarily fall under specialists for diagnosis and prescription. For “3.2.1 Dietary management” and “3.2.2 Exercise management,” the experts advised simplifying the language to improve clarity.

In “3.4 Self-monitoring,” it was suggested to merge “3.4.3 Identifying and managing hypoglycemia” with “3.4.2 Self-blood glucose monitoring” due to overlapping content. Additionally, the experts recommended adding “self-monitoring of urine status” to this section. Lastly, the experts proposed merging “3.5.3 Providing information on public health service policies to patients and their families” with “3.5.4 Informing patients and their families about diabetes health management services available at primary healthcare institutions” to streamline content.

#### 3.3.4 Final protocol establishment

During the second round of expert consultation, one expert suggested adding indications for “3.3.1 Timed voiding” and “3.3.2 Delayed voiding.” Following discussions, the panel reached a consensus on a management protocol comprising four primary indicators, 17 secondary indicators, and 60 tertiary indicators.

#### 3.3.5 Weights of various indicators

**Figure 3:**
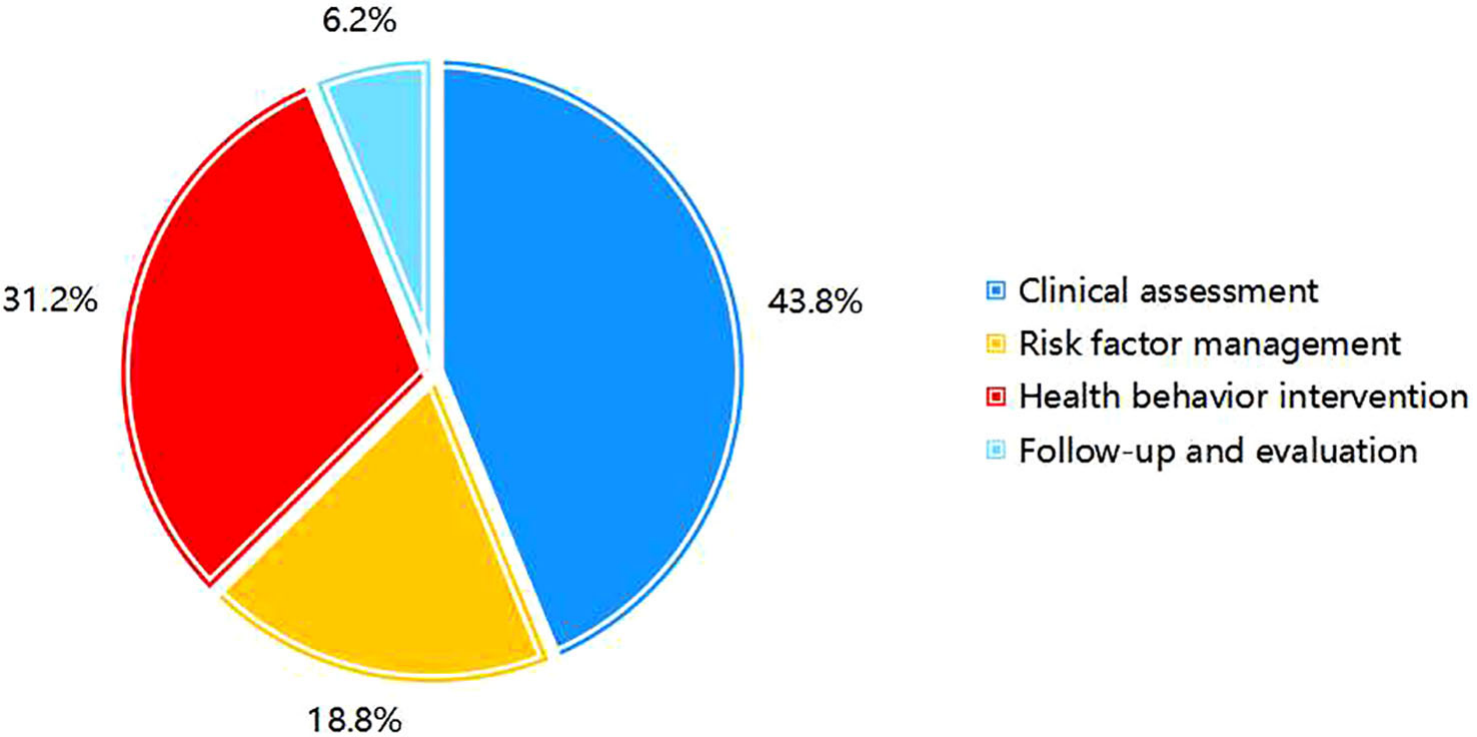
Weight distribution of primary indicators.

**Figure 4:**
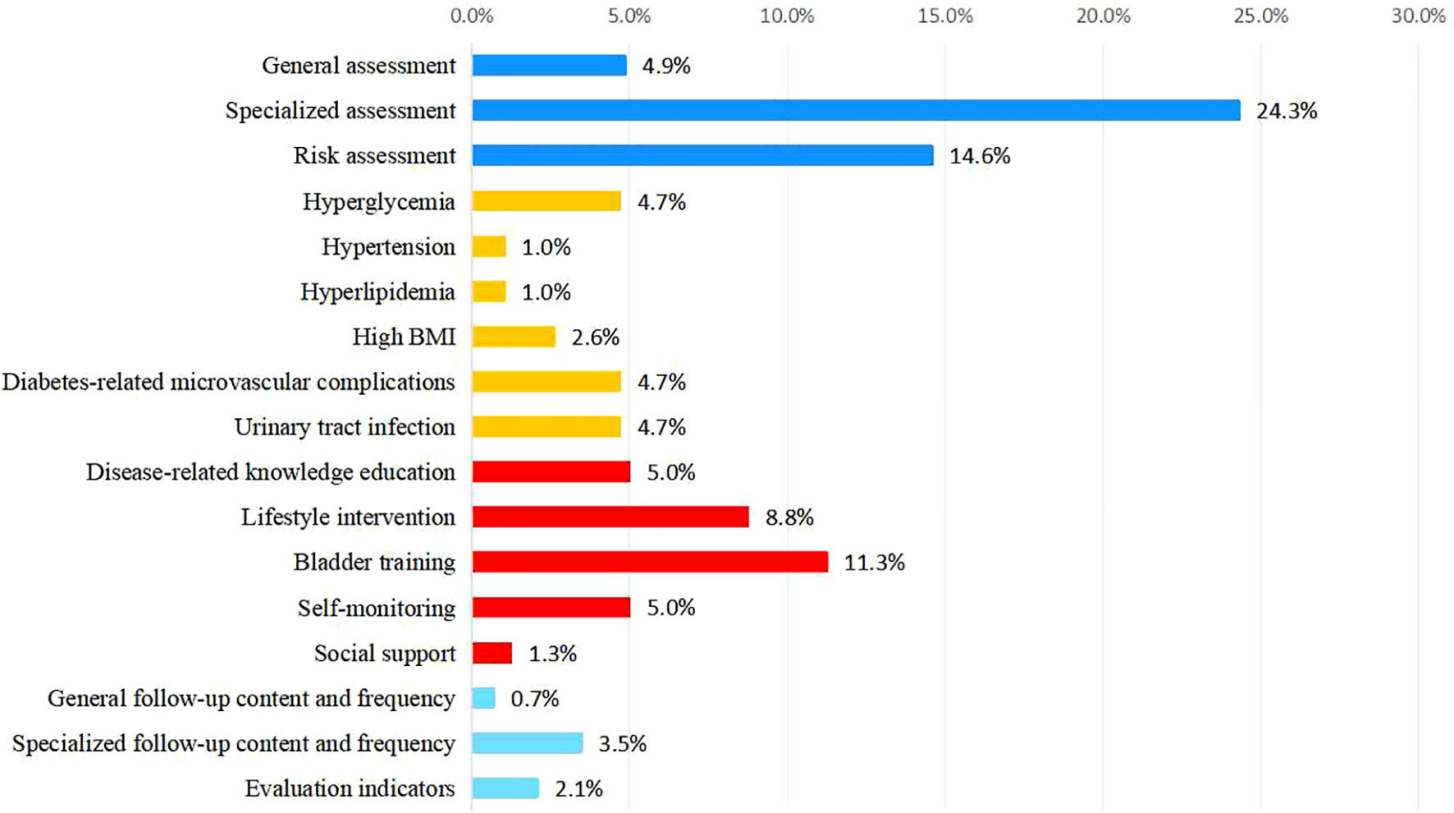
Weight distribution of secondary indicators.

Among the primary indicators, “Clinical assessment” holds the highest weight at 0.438. Among the secondary indicators, “Specialized assessment” was the most significant, with a weight of 0.243, followed by “Bladder function training” at 0.113. For the tertiary indicators, “Symptom assessment” ranked highest, with a weight of 0.106 (**Figures 3**, **4**). Selected statistical data are presented in **Table 4**, with comprehensive details available in **Supplementary Material 1**.

**Table 4:** Statistical summary of primary and secondary indicators: mean, SD, CV, full score ratio, and weight.

Indicators	Mean	SD	CV	Full score ratio	Weight
1. Clinical assessment	4.94	0.24	0.05	0.94	0.438
1.1 General assessment	4.78	0.43	0.09	0.78	0.049
1.2 Specialized assessment	4.94	0.24	0.05	0.94	0.243
1.3 Risk assessment	4.83	0.38	0.08	0.83	0.146
2. Risk factor management	4.56	0.78	0.17	0.78	0.188
2.1 Hyperglycemia	4.94	0.24	0.05	0.94	0.047
2.2 Hypertension	4.67	0.59	0.13	0.72	0.010
2.3 Hyperlipidemia	4.67	0.59	0.13	0.72	0.010
2.4 High BMI	4.72	0.57	0.12	0.78	0.026
2.5 Diabetes-related microvascular complications (diabetic neuropathy, diabetic retinopathy (DR), and diabetic nephropathy (DN)).	4.94	0.24	0.05	0.94	0.047
2.6 Urinary tract infection	4.94	0.24	0.05	0.94	0.047
3. Health behavior intervention	4.89	0.32	0.07	0.89	0.312
3.1 Disease-related knowledge education	4.78	0.43	0.09	0.78	0.050
3.2 Lifestyle intervention	4.83	0.38	0.08	0.83	0.088
3.3 Bladder training	4.89	0.32	0.07	0.89	0.113
3.4 Self-monitoring	4.78	0.43	0.09	0.78	0.050
3.5 Social support	0.50	0.51	0.11	0.50	0.013
4. Follow-up and evaluation	4.33	0.77	0.18	0.56	0.062
4.1 General follow-up content and frequency	4.67	0.49	0.10	0.67	0.007
4.2 Specialized follow-up content and frequency	4.83	0.38	0.08	0.83	0.035
4.3 Evaluation indicators	4.78	0.43	0.09	0.78	0.021

SD, standard deviation; CV, coefficient of variation.

## 4 Discussion

This Delphi consensus study established an evidence-based clinical framework for managing neurogenic bladder (NB) risk in Chinese patients with T2DM. Through two iterative rounds of expert consultation, we developed a management protocol for DNB, structured hierarchically with 4 first-level, 17 second-level, and 60 third-level indicators.

The findings demonstrate substantial consistency in expert consensus, with 19 specialists from four Chinese geographical regions contributing multidisciplinary perspectives (urology, endocrinology, rehabilitation, and health management). The consultation process achieved Cr values of 0.894 and 0.914 in successive rounds, reflecting participants’ robust theoretical and practical expertise in DNB management. However, the statistically significant yet weak Kendall’s *W* value reveals an inherent tension: while the experts assigned uniformly high importance ratings, limiting differentiation among the indicators, their diverse disciplinary backgrounds and varying weighting criteria resulted in only moderate concordance. This paradox, characterized by significant yet weak agreement, highlights a recognized limitation of Delphi methodologies involving heterogeneous expert panels (36).

The protocol’s principal innovation lies in synthesizing existing evidence through Delphi consensus to establish China’s first comprehensive management framework for high-risk NB in patients with T2DM. The protocol consists of four core components: clinical assessment, risk factor management, health behavior intervention, and follow-up evaluation.

Our protocol markedly differs from conventional DNB management, which primarily focuses on isolated interventions. Traditional strategies predominantly target urological symptom management through anticholinergic medications or intermittent catheterization (46), typically initiated only after detrusor dysfunction has developed. In contrast, our approach emphasizes primary prevention via early risk stratification using a novel prediction model (14) that incorporates age, diabetic peripheral neuropathy (DPN), glycated hemoglobin (HbA1c), and absolute neutrophil count (ANC). This model (14) achieved good predictive performance (AUC=0.817). A patient-specific risk score was calculated for each participant; those scoring above a predefined threshold (yielding a sensitivity of 88.1% and specificity of 50.0%) were enrolled. Thereby, it enables early identification of high-risk patients before irreversible bladder damage occurs (14).

The health behavior intervention component integrates the Integrated Theory of Health Behavior Change (ITHBC) (47) with China’s sociocultural context through three key adaptations. First, dietary recommendations accommodate traditional Chinese preferences while restricting bladder irritants like caffeine—an essential adjustment given that Chinese patients have historically prioritized medical treatments over dietary modifications (48, 49). Second, Wuyin music therapy, rooted in traditional Chinese medicine, is incorporated to address diabetes-related stress (50), building on existing evidence supporting music interventions for chronic disease management (51, 52). Third, a multi-tiered support system engages families, clinicians, and peers, recognizing the Chinese cultural emphasis on collective health behaviors. Randomized trials have demonstrated that family-assisted interventions result in 40% greater compliance compared to individual approaches (53).

This study employed the Delphi method to identify individuals at high risk of DNB among Chinese patients with T2DM, and to formulate a consensus on non-pharmacological interventions for delaying or preventing DNB onset. In parallel, conventional pharmacological management and the prevention of recurrent urinary tract infections (UTIs) remain critical. Evidence indicates that patients with T2DM are at a substantially elevated risk of both UTIs and recurrent UTIs compared to non-diabetic populations (54, 55). Regular screening for UTI-related symptoms is therefore recommended in this cohort (54). Regarding glucose-lowering agents, glucagon-like peptide-1 receptor agonists (GLP-1RAs) are recommended as first-line therapy to mitigate overall infection risk (56). If sodium-glucose cotransporter-2 (SGLT-2) inhibitors are indicated, vigilant assessment and continuous monitoring for UTI signs are imperative (57). Antimicrobial strategies should be individualized, with avoidance of unnecessary prophylactic antibiotics; however, enhanced surveillance and management are warranted in high-risk subgroups, such as those with recurrent UTIs or underlying urinary tract anatomical abnormalities (54).

It is crucial to note that the findings and recommendations herein specifically pertain to NB precipitated by diabetes within the T2DM population. This study focuses on high-risk, diabetes-precipitated NB patients. In contrast, NB predating diabetes and subsequently followed by incident diabetes constitutes an independent etiologic and clinical trajectory. The two scenarios differ fundamentally in their etiopathogenesis, temporal progression, and implications for clinical management, and are not directly interchangeable for extrapolation. Accordingly, conclusions derived from diabetes-precipitated NB should not be indiscriminately generalized to NB in the context of incident diabetes. Clear etiologic differentiation is essential to guide context-appropriate care, underscoring the need for future comparative research.

While the protocol offers systematic guidance for DNB management, its effectiveness requires empirical validation. The inherent reliance of the Delphi method on expert consensus introduces potential subjectivity; however, this was mitigated through predefined consensus thresholds and panel diversity. While a substantial consensus has been established among multidisciplinary experts in this study, targeted efforts are required to strengthen this alignment further. Additionally, the protocol’s specificity to the Chinese healthcare context may limit its generalizability, necessitating adaptation studies for broader populations. Implementation challenges, including resource constraints and variations in clinician expertise, should be further explored through planned randomized controlled trials.

## 5 Conclusion

In conclusion, we used the Delphi method to develop a risk management protocol for NB in patients with T2DM. This protocol establishes a standardized approach for the early management of those at high risk, ensuring a comprehensive and evidence-based framework. Its authoritative and reliable content provides a strong theoretical foundation for enhancing NB risk management in individuals with T2DM in China while offering valuable guidance for future clinical practice and research.

## Data Availability

The raw data supporting the conclusions of this article will be made available by the authors, without undue reservation.
