# Supplementary material for: Construction of a management protocol for high-risk neurogenic bladder in Chinese patients with type 2 diabetes: a Delphi study
Source: Front Endocrinol (Lausanne). 2025 Oct 17;16:1603905. doi: 10.3389/fendo.2025.1603905 (PMC12541250; doi:10.3389/fendo.2025.1603905)
Supplement: Supplementary file 1 [file DataSheet1.doc]

**Supplementary Material**

****《**Construction of a Management Protocol for High-Risk Neurogenic Bladder in Chinese Patients with Type 2 Diabetes: A Delphi Study》Expert consultation form（first round）**

**Dear Expert,**

**It is a great honor to invite you as a consulting expert for this research project. Your extensive experience and profound expertise are crucial for the development of this project.Thank you for your participation.**

**Diabetic Neurogenic Bladder (DNB), also known as diabetic bladder dysfunction, is a lower urinary tract dysfunction caused by disturbances in the neural control mechanisms in patients with diabetes. We hope to provide patients with proper and effective bladder care through a scientific and standardized management program, improve compliance, and ultimately enhance outcomes. In our preliminary research, we established a DNB risk prediction model and initially drafted a DNB risk management program based on evidence, literature review, and group discussions. Our research team aims to refine the initially proposed management program using expert consultation to construct a scientifically feasible intervention program for the prevention of DNB.**

**In the later stages, we will apply the DNB risk management program based on the prediction model in clinical settings to provide standardized and scientific guidance for the prevention of DNB. The initially proposed management program includes 4 primary indicators, 15 secondary indicators, and 55 tertiary indicators. We kindly ask you to evaluate the initial intervention program, and please feel free to correct any inappropriate aspects. Due to the needs of the research project, we would appreciate it if you could return the consultation form within 7 days. If you have any questions regarding the issues addressed in the survey, we warmly welcome and appreciate your contact with us.**

**We greatly appreciate your support and collaboration in this important endeavor.**

**Contact information：Senying Luo**

**Tel：+8613632761081 E-Mail：**[173121623@qq.com](mailto:173121623@qq.com)

**Part 1:Expert Information Questionnaire**

**This section of the survey is for research analysis purposes only and will be strictly kept confidential. Please fill in your basic personal information or mark “√” on the appropriate options. If you need to provide explanations, please indicate them in the corresponding section.**

| **Age** |  | **Gender** | ☐Male ☐Female | **Years of experience** |  |
| --- | --- | --- | --- | --- | --- |
| **Organization** |  | | | **Position** |  |
| **Academic qualification** | ☐Senior ☐Associate Senior ☐Intermediate ☐Other | | | | |
| **Educational background** | ☐Doctorate ☐Master’s degree ☐Bachelor's degree ☐Associate degree | | | | |
| **Professional area of expertise** | ☐Nursing ☐Endocrinology ☐Urology ☐Rehabilitation ☐Health management | | | | |
| **Contact information** | ☐Telephone number____________________ ☐E-mail_______________ ☐Other ___________________ | | | | |
| **Familiarity with the field** | ☐Very familiar ☐Fairly familiar ☐Average ☐Unfamiliar ☐Quite unfamiliar | | | | |
| **Judgement of experts regarding the field** | **Criteria** | **Expert Self-assessment** | | | |
|  | Experience | ☐High ☐Medium ☐Low | | | |
|  | Theoretical Analysis | ☐High ☐Medium ☐Low | | | |
|  | Refer to domestic and international literature | ☐High ☐Medium ☐Low | | | |
|  | Intuitive choice | ☐High ☐Medium ☐Low | | | |

**Part Two: Expert Consultation Questionnaire**

****Instructions:****Table 1 and Table 2 represent the primary and secondary indicators of the "**Risk management program for neurogenic bladder in type 2 diabetes mellitus** consultation form." Please rate the importance of each item using a Likert 5-point scale, where "very important (5 points) to very unimportant (1 point)." Please mark "√" in the corresponding position. If you have any suggestions for modifications regarding the scale dimensions and items, please fill them in the "Modification Suggestions" column. If you believe there are dimensions or items that can be added or removed, please indicate them in the "Add or Remove" column and provide an importance rating for any dimensions or items that need to be added.

**Table 1 Construction of a Risk Management Program for Neurogenic Bladder in Patients with Type 2 Diabetes Mellitus**

**（First-level indicator evaluation form）**

| **First-level indicator** | **Importance rating** | | | | | **Modification suggestions** |
| --- | --- | --- | --- | --- | --- | --- |
|  | **5** | **4** | **3** | **2** | **1** |  |
| 1.Assessment | **☐** | **☐** | **☐** | **☐** | **☐** |  |
| 2.Risk factor management | **☐** | **☐** | **☐** | **☐** | **☐** |  |
| 3.Health behavior change intervention | **☐** | **☐** | **☐** | **☐** | **☐** |  |
| 4.Follow-up and evaluation | **☐** | **☐** | **☐** | **☐** | **☐** |  |
| **Add dimension** |  | | | | | |
| **Remove dimension** |  | | | | | |

**Table 2 Construction of a Risk Management Program for Neurogenic Bladder in Patients with Type 2 Diabetes Mellitus**

**（Second-level indicator evaluation form）**

| **First-level indicator** | **Second-level indicator** | **Importance rating** | | | | | **Modification suggestions** |
| --- | --- | --- | --- | --- | --- | --- | --- |
|  |  | **5** | **4** | **3** | **2** | **1** |  |
| 1.Assessment | 1.1 General assessment | ☐ | ☐ | ☐ | ☐ | ☐ |  |
|  | 1.2 Specialized assessment | ☐ | ☐ | ☐ | ☐ | ☐ |  |
|  | 1.3 Risk assessment | ☐ | ☐ | ☐ | ☐ | ☐ |  |
| **Add item** |  | | | | | | |
| **Remove item** |  | | | | | | |
| 2.Risk factor management | 2.1 Hyperglycemia | ☐ | ☐ | ☐ | ☐ | ☐ |  |
|  | 2.2 Hypertension | ☐ | ☐ | ☐ | ☐ | ☐ |  |
|  | 2.3 Hyperlipidemia | ☐ | ☐ | ☐ | ☐ | ☐ |  |
|  | 2.4 Renal function abnormalities | ☐ | ☐ | ☐ | ☐ | ☐ |  |
|  | 2.5 Peripheral neuropathy | ☐ | ☐ | ☐ | ☐ | ☐ |  |
| **Add item** |  | | | | | | |
| **Remove item** |  | | | | | | |
| 3.Health behavior change intervention | 3.1 Knowledge and belief education | ☐ | ☐ | ☐ | ☐ | ☐ |  |
|  | 3.2 Lifestyle intervention | ☐ | ☐ | ☐ | ☐ | ☐ |  |
|  | 3.3 Bladder training | ☐ | ☐ | ☐ | ☐ | ☐ |  |
|  | 3.4 Self-monitoring | ☐ | ☐ | ☐ | ☐ | ☐ |  |
|  | 3.5 Social support | ☐ | ☐ | ☐ | ☐ | ☐ |  |
| **Add item** |  | | | | | | |
| **Remove item** |  | | | | | | |
| 4.Follow-up and evaluation | 4.1 Follow-up content and frequency | ☐ | ☐ | ☐ | ☐ | ☐ |  |
|  | 4.2 Evaluation indicators | ☐ | ☐ | ☐ | ☐ | ☐ |  |
| **Add item** |  | | | | | | |
| **Remove item** |  | | | | | | |

**Table 3 Construction of a Risk Management Program for Neurogenic Bladder in Patients with Type 2 Diabetes Mellitus**

**（Third-level indicator evaluation form）**

| **First-level indicator** | **Second-level indicator** | **Third-level indicator** | **Importance rating** | | | | | **Modification suggestions** |
| --- | --- | --- | --- | --- | --- | --- | --- | --- |
|  |  |  | **5** | **4** | **3** | **2** | **1** |  |
| 1.Assessment | 1.1 Clinical assessment | 1.1.1The endocrinologist conducts an assessment of the medical history, physical examination, auxiliary examination , and medication status | ☐ | ☐ | ☐ | ☐ | ☐ |  |
|  |  | 1.1.2 Lifestyle assessment | ☐ | ☐ | ☐ | ☐ | ☐ |  |
|  | 1.2 Specialized assessment | 1.2.1 Neuropathy assessment | ☐ | ☐ | ☐ | ☐ | ☐ |  |
|  |  | 1.2.2 Symptom assessment: including lower urinary tract symptoms, bladder sensory abnormalities, neurological symptoms, gastrointestinal symptoms, and other symptom | ☐ | ☐ | ☐ | ☐ | ☐ |  |
|  |  | 1.2.3 Urodynamic or imaging urodynamic assessment |  |  |  |  |  |  |
|  | 1.3 Risk assessment | 1.3.1 Are the following risk factors present: hyperglycemia, hypertension, hyperlipidemia, peripheral neuropathy, renal function abnormalities | ☐ | ☐ | ☐ | ☐ | ☐ |  |
|  |  | 1.3.2 Use of risk prediction model (based on the previous research of the research group) (Logit(P) = -7.574 + 0.063 × Age + 1.491 × Urinary Microalbumin-to-Creatinine Ratio + 0.127 × Serum Urea + 0.183 × Absolute Neutrophil Count) for assessment, with a cutoff value of >0.367 indicating high-risk patients | ☐ | ☐ | ☐ | ☐ | ☐ |  |
| 2.Risk factor management | 2.1 Hyperglycemia | 2.1.1 Controlling hyperglycemia includes five major measures: medical nutrition therapy, exercise therapy, blood glucose monitoring, diabetes education, and the use of hypoglycemic medications. The hypoglycemic regimen follows the strategies recommended in the ‘Guidelines for the Prevention and Treatment of Type 2 Diabetes in China 2020’ | ☐ | ☐ | ☐ | ☐ | ☐ |  |
|  |  | 2.1.2 The blood glucose control targets adhere to the recommended values in the 'Guidelines for the Prevention and Treatment of Type 2 Diabetes in China 2020,' HbA1c of general adult < 7.0% and HbA1c of elderly ≤ 8% | ☐ | ☐ | ☐ | ☐ | ☐ |  |
|  |  | 2.1.3 If blood glucose levels are not achieved through lifestyle interventions alone, pharmacological treatment should be initiated. | ☐ | ☐ | ☐ | ☐ | ☐ |  |
|  | 2.2 Hypertension | 2.2.1 Patients should monitor their blood pressure daily at home. | ☐ | ☐ | ☐ | ☐ | ☐ |  |
|  |  | 2.2.2 Strengthen blood pressure control, with individualized blood pressure targets. The general target for blood pressure reduction in diabetic patients is < 130/80 mmHg. | ☐ | ☐ | ☐ | ☐ | ☐ |  |
|  |  | 2.2.3 Non-pharmacological treatment: Lifestyle interventions should begin when the patient's blood pressure level is > 120/80 mmHg, as stated in section 3.2. | ☐ | ☐ | ☐ | ☐ | ☐ |  |
|  |  | 2.2.4 Antihypertensive medication treatment: Consider initiating antihypertensive medication when the patient's blood pressure is ≥ 140/90 mmHg. The antihypertensive strategy follows the 'ADA Standards of Medical Care in Diabetes (2024).’ | ☐ | ☐ | ☐ | ☐ | ☐ |  |
|  | 2.3 Hyperlipidemia | 2.3.1 Optimize lipid management: Low-density lipoprotein cholesterol (LDL-C) and non-high-density lipoprotein cholesterol (non-HDL-C) are primary targets for lipid intervention in diabetic patients. The lipid control targets follow the recommended values in the ‘Chinese Expert Consensus on Lipid Management in Diabetic Patients 2024.' | ☐ | ☐ | ☐ | ☐ | ☐ |  |
|  |  | 2.3.2 Lifestyle interventions, as stated in section 3.2 | ☐ | ☐ | ☐ | ☐ | ☐ |  |
|  |  | 2.3.3 The pharmacological lipid-lowering treatment strategy follows the 'Chinese Expert Consensus on Lipid Management in Diabetic Patients 2024' | ☐ | ☐ | ☐ | ☐ | ☐ |  |
|  | 2.4 Renal function abnormalities | 2.4.1 Strengthen blood glucose control, as stated in section 2.1. | ☐ | ☐ | ☐ | ☐ | ☐ |  |
|  |  | 2.4.2 Gradually improve blood glucose: Changes in HbA1c should not exceed 3 points within 3 months to avoid inducing 'treatment-induced diabetic neuropathy | ☐ | ☐ | ☐ | ☐ | ☐ |  |
|  |  | 2.4.3 Optimize blood pressure and lipid management, as stated in sections 2.2 and 2.3. | ☐ | ☐ | ☐ | ☐ | ☐ |  |
|  |  | 2.4.4 Discontinue medications that may cause or exacerbate neuropathy | ☐ | ☐ | ☐ | ☐ | ☐ |  |
|  |  | 2.4.5 Guide patients on proper foot care | ☐ | ☐ | ☐ | ☐ | ☐ |  |
|  | 2.5 Peripheral neuropathy | 2.5.1 Monitor kidney function every 3 to 6 months | ☐ | ☐ | ☐ | ☐ | ☐ |  |
|  |  | 2.5.2 For patients using oral hypoglycemic agents, adjust the dosage of these medications based on the estimated glomerular filtration rate (eGFR) | ☐ | ☐ | ☐ | ☐ | ☐ |  |
|  |  | 2.5.3 Avoid using medications that can cause kidney damage | ☐ | ☐ | ☐ | ☐ | ☐ |  |
|  |  | 2.5.4 If a patient's urinary albumin levels remain elevated and/or eGFR continues to decline and/or eGFR is <30 mL/min/1.73 m², refer to a nephrologist. | ☐ | ☐ | ☐ | ☐ | ☐ |  |
| 3.Health behavior change intervention | 3.1 Knowledge and belief education | 3.1.1 Guide patients and their families to learn about diabetes, diabetic neurogenic bladder, and bladder management knowledge and skills. | ☐ | ☐ | ☐ | ☐ | ☐ |  |
|  |  | 3.1.2 Educate patients and their families about the adverse outcomes of diabetic neurogenic bladder and the benefits of early bladder management. | ☐ | ☐ | ☐ | ☐ | ☐ |  |
|  | 3.2 Lifestyle intervention | 3.2.1 Nutritional Management  (1) Develop individualized dietary prescriptions based on the patient's age, height, weight, metabolic indicators, and organ function, calculating energy intake according to (25~30 kcal)·kg⁻¹ (standard weight)·d⁻¹, following the recommended treatment strategies outlined in the "Chinese Guidelines for the Prevention and Treatment of Type 2 Diabetes" (2020).  (2) Maintain a body mass index (BMI) of less than 24 kg/m².  (3) Ensure a daily water intake of 1500-2000 ml, reducing water intake at night to avoid large amounts of drinking in a short period.  (4) For patients with hypertension: adopt the Dietary Approaches to Stop Hypertension (DASH) diet, reducing sodium intake to less than 2.3 g/d.  (5) For patients with hyperlipidemia: adopt the Mediterranean diet or the DASH diet, limiting total fat, saturated fatty acids, cholesterol, and trans fatty acids intake.  (6) For patients with renal dysfunction: for those not undergoing dialysis, protein intake should be 0.8 g·kg⁻¹·d⁻¹, and for those undergoing dialysis, protein intake should be 1g·kg⁻¹·d⁻¹. | ☐ | ☐ | ☐ | ☐ | ☐ |  |
|  |  | 3.2.2 Exercise Management  (1) Engage in low-intensity aerobic exercise for 5-7 days a week, ideally exercising every day. The best time to exercise is 1 hour after meals, with approximately 20 minutes of exercise after each meal.  (2) Perform resistance training 2-3 times a week, with each session consisting of 1-3 sets of exercises, and each set/exercise repeated 10-15 times.  (3) Core muscle training. | ☐ | ☐ | ☐ | ☐ | ☐ |  |
|  |  | 3.2.3 Sleep Management  (1) Maintain a regular sleep schedule. (2) Aim for 6-8 hours of sleep each night. (3) Create a conducive sleep environment (dark, quiet, with appropriate temperature and humidity). (4) Refer patients with significant sleep difficulties to a sleep specialist. | ☐ | ☐ | ☐ | ☐ | ☐ |  |
|  |  | 3.2.4 Stress Management  (1) Teach patients relaxation techniques and mindfulness meditation for self-relaxation. (2) Use sound therapy to regulate emotions by listening to pentatonic music after meals, five times a week for 30 minutes each session. (3) Refer patients with psychological issues to a mental health specialist for treatment. | ☐ | ☐ | ☐ | ☐ | ☐ |  |
|  |  | 3.2.5 Avoid Harmful Substances and Behaviors  (1) Quit smoking (2) Abstain from alcohol; if drinking, avoid doing so on an empty stomach. (3) Avoid staying up late (4) Avoid wearing clothing that causes skin allergies, tight-fitting garments, and sitting baths. | ☐ | ☐ | ☐ | ☐ | ☐ |  |
|  |  | 3.2.6 Interpersonal Relationships: Establish good relationships with family, friends, patients, and the healthcare team to assist in disease management. | ☐ | ☐ | ☐ | ☐ | ☐ |  |
|  | 3.3 bladder training | 3.3.1 Pelvic Floor Muscle Training (PFMT). | ☐ | ☐ | ☐ | ☐ | ☐ |  |
|  |  | 3.3.2 Timed voiding. | ☐ | ☐ | ☐ | ☐ | ☐ |  |
|  |  | 3.3.3Delayed voiding. | ☐ | ☐ | ☐ | ☐ | ☐ |  |
|  | 3.4 self-monitoring | 3.4.1 Bladder Diary  (1) Record drinking times, drinking amounts, urination times, urination amounts, and accompanying symptoms.  (2) Continuously record for 3 days. | ☐ | ☐ | ☐ | ☐ | ☐ |  |
|  |  | 3.4.2 Self-Monitoring of Blood Glucose: It can be scheduled for fasting, after three meals, before bedtime, or before and after vigorous exercise. | ☐ | ☐ | ☐ | ☐ | ☐ |  |
|  |  | 3.4.3 Identifying and Managing Hypoglycemia: Keep carbohydrate-rich foods on hand; consume them immediately if hypoglycemia occurs. | ☐ | ☐ | ☐ | ☐ | ☐ |  |
|  | 3.5 social support | 3.5.1 Encourage family and friends to provide emotional support to the patient. | ☐ | ☐ | ☐ | ☐ | ☐ |  |
|  |  | 3.5.2 Achieve peer support through diabetes clubs and the use of the internet. | ☐ | ☐ | ☐ | ☐ | ☐ |  |
|  |  | 3.5.3 Provide patients and their families with information related to public health service policies. | ☐ | ☐ | ☐ | ☐ | ☐ |  |
|  |  | 3.5.4 Inform patients and their families that primary healthcare institutions provide diabetes health management services. | ☐ | ☐ | ☐ | ☐ | ☐ |  |
|  |  | 3.5.5 Provide patients and their families with information related to medical insurance. | ☐ | ☐ | ☐ | ☐ | ☐ |  |
| **Add item** |  | | | | | | | |
| **Remove item** |  | | | | | | | |
| 4.Follow-up and evaluation | 4.1 Follow-up content and frequency | 4.1.1 Monitor BMI, waist circumference, blood pressure, and blood glucose monthly. | ☐ | ☐ | ☐ | ☐ | ☐ |  |
|  |  | 4.1.2 Test HbA1c every three months until the target is reached, and every six months thereafter. | ☐ | ☐ | ☐ | ☐ | ☐ |  |
|  |  | 4.1.3 Conduct urinalysis once every two months. | ☐ | ☐ | ☐ | ☐ | ☐ |  |
|  |  | 4.1.4 Kidney function tests: Conduct annually for routine checks, and every 3-6 months for those with abnormal kidney function. | ☐ | ☐ | ☐ | ☐ | ☐ |  |
|  |  | 4.1.5 Perform urinary system ultrasound and residual urine volume measurement once every six months. | ☐ | ☐ | ☐ | ☐ | ☐ |  |
|  |  | 4.1.6 Conduct urodynamic tests once a year. | ☐ | ☐ | ☐ | ☐ | ☐ |  |
|  |  | 4.1.7 It is recommended to use imaging urodynamic testing; if not feasible, asynchronous bladder-urethra contrast imaging combined with urodynamic testing should be performed. | ☐ | ☐ | ☐ | ☐ | ☐ |  |
|  |  | 4.1.8 If the patient experiences discomfort or notices abnormalities in urine color or characteristics, they should seek medical attention promptly. | ☐ | ☐ | ☐ | ☐ | ☐ |  |
|  | 4.2 Evaluation indicators | 4.2.1Primary outcome indicators: bladder residual urine volume, Neurogenic Bladder Symptom Score (NBSS) | ☐ | ☐ | ☐ | ☐ | ☐ |  |
|  |  | 4.2.2Secondary outcome indicators: metabolic indicators (HbA1c, blood pressure, blood lipids), Diabetes Self-Management Questionnaire (DSMQ) score | ☐ | ☐ | ☐ | ☐ | ☐ |  |
| **Add item** |  | | | | | | | |
| **Remove item** |  | | | | | | | |
