# Supplementary material for: Construction of a management protocol for high-risk neurogenic bladder in Chinese patients with type 2 diabetes: a Delphi study
Source: Front Endocrinol (Lausanne). 2025 Oct 17;16:1603905. doi: 10.3389/fendo.2025.1603905 (PMC12541250; doi:10.3389/fendo.2025.1603905)
Supplement: Supplementary file 2 [file DataSheet2.docx]

Table S1 Statistical Summary of Indicators: Mean, SD, CV, Full Score Ratio, and Weight

| **Indicators** | **Mean** | **SD** | **CV** | **Full score ratio** | **Weight** |
| --- | --- | --- | --- | --- | --- |
| 1. **Clinical assessment** | 4.94 | 0.24 | 0.05 | 0.94 | 0.438 |
| **1.1 General assessment** | 4.78 | 0.43 | 0.09 | 0.78 | 0.049 |
| 1.1.1 Medical history: age, gender, duration of diabetes, family history, past medical history (Benign Prostatic Hyperplasia, History of Urinary Tract Infections), medication history. | 4.89 | 0.32 | 0.07 | 0.89 | 0.014 |
| 1.1.2 Physical examination: measure blood pressure, heart rate, height, weight, waist circumference, hip circumference, and calculate BMI and waist-to-hip ratio. | 4.72 | 0.46 | 0.10 | 0.72 | 0.006 |
| 1.1.3 Laboratory tests: Glycated Hemoglobin (HbA1c), Complete Blood Count, Lipid Profile, liver function tests, kidney function tests, serum electrolytes, urinalysis, Urine Albumin/Creatinine Ratio. | 4.83 | 0.38 | 0.08 | 0.83 | 0.010 |
| 1.1.4 Complication screening: Diabetic Neuropathy, Diabetic Retinopathy, Diabetic Nephropathy. | 5.00 | 0.00 | 0.00 | 1.00 | 0.017 |
| 1.1.5 Lifestyle assessment: Including smoking, alcohol consumption, physical activity, diet, sleep, stress, and social relationships. | 4.67 | 0.49 | 0.10 | 0.67 | 0.002 |
| **1.2 Specialized assessment** | 4.94 | 0.24 | 0.05 | 0.94 | 0.243 |
| 1.2.1 Symptom assessment (according to the 2024 European Urology Association Guidelines) (1) Urinary symptoms (2) Sexual dysfunction symptoms (3) Gastrointestinal symptoms (4) Neurological symptoms (5) Other symptoms (including fever, low back pain, pelvic pain, hematuria, pyuria) | 5.00 | 0.00 | 0.00 | 1.00 | 0.106 |
| 1.2.2 Management methods for the urinary system: spontaneous urination, Valsalva Maneuver, tapping for urination, compression for urination, involuntary urinary leakage, Intermittent Catheterization, Long-term Indwelling Catheterization, and indwelling bladder stoma. | 4.83 | 0.38 | 0.08 | 0.72 | 0.015 |
| 1.2.3 Physical examination of the urogenital and nervous systems (European Urology Association 2024 Guidelines): including urogenital sensation and reflexes, anal sphincter function, and pelvic floor function. | 4.94 | 0.24 | 0.05 | 0.94 | 0.061 |
| 1.2.4 Urodynamic Testing: Residual Urine Measurement, Imaging Urodynamics. | 4.94 | 0.24 | 0.05 | 0.94 | 0.061 |
| **1.3 Risk assessment** | 4.83 | 0.38 | 0.08 | 0.83 | 0.146 |
| 1.3.1 Are there any of the following risk factors: age, high BMI, duration of diabetes, high glycated hemoglobin, hypertension, hyperlipidemia, diabetic neuropathy, diabetic retinopathy, diabetic nephropathy, urinary tract infection? | 4.78 | 0.43 | 0.09 | 0.78 | 0.109 |
| 1.3.2 Use of risk prediction model (based on the previous research of the research group) (Logit(P) = -7.574 + 0.063 × Age + 1.491 × Urinary Microalbumin-to-Creatinine Ratio + 0.127 × Serum Urea + 0.183 × Absolute Neutrophil Count) for assessment, with a cutoff value of >0.367 indicatinghigh-risk patients | 4.67 | 0.49 | 0.10 | 0.72 | 0.036 |
| **2.Risk factor management** | 4.56 | 0.78 | 0.17 | 0.78 | 0.188 |
| **2.1 Hyperglycemia** | 4.94 | 0.24 | 0.05 | 0.94 | 0.047 |
| 2.1.1 HbA1c target values: general patients < 7.0%, elderly patients ≤ 8%. | 4.78 | 0.43 | 0.09 | 0.78 | 0.035 |
| 2.1.2 Time in Range (TIR) control targets for glucose: general patients > 70%, elderly patients > 50%. | 4.61 | 0.98 | 0.21 | 0.78 | 0.012 |
| **2.2 Hypertension** | 4.67 | 0.59 | 0.13 | 0.72 | 0.010 |
| 2.2.1 Patients should monitor their blood pressure daily at home. | 4.56 | 0.62 | 0.14 | 0.61 | 0.005 |
| 2.2.2 Enhanced blood pressure control: The target blood pressure for general diabetes patients is < 130/80 mmHg. | 4.56 | 0.62 | 0.14 | 0.61 | 0.005 |
| **2.3 Hyperlipidemia** | 4.67 | 0.59 | 0.13 | 0.72 | 0.010 |
| 2.3.1 For patients with concomitant Atherosclerotic Cardiovascular Disease (ASCVD), the target Low-Density Lipoprotein Cholesterol (LDL-C) level is < 1.4 mmol/L. | 4.67 | 0.49 | 0.10 | 0.67 | 0.006 |
| 2.3.2 For high-risk patients with ASCVD, the target Low-Density Lipoprotein Cholesterol (LDL-C) level is < 1.8 mmol/L. | 4.61 | 0.61 | 0.13 | 0.67 | 0.002 |
| 2.3.3 For other patients, the target Low-Density Lipoprotein Cholesterol (LDL-C) level is < 2.6 mmol/L. | 4.61 | 0.61 | 0.13 | 0.67 | 0.002 |
| **2.4 High BMI** | 4.72 | 0.57 | 0.12 | 0.78 | 0.026 |
| 2.4.1 Maintain body mass index (BMI) < 24 kg/m². | 4.56 | 0.62 | 0.14 | 0.61 | 0.007 |
| 2.4.2 Overweight or obese patients should aim for a weight loss of 5%. | 4.61 | 0.50 | 0.11 | 0.61 | 0.020 |
| 2.5 Diabetes-related microvascular complications (diabetic neuropathy, diabetic retinopathy (DR), diabetic nephropathy (DN)). | 4.94 | 0.24 | 0.05 | 0.94 | 0.047 |
| 2.5.1 Strengthen blood glucose control and optimize blood pressure and lipid management, as outlined in sections 2.1, 2.2, and 2.3. | 4.94 | 0.24 | 0.05 | 0.94 | 0.011 |
| 2.5.2 Gradually improve blood glucose levels: Changes in glycated hemoglobin should not exceed 3 percentage points within 3 months, to avoid inducing "treatment-induced diabetic neuropathy". | 4.89 | 0.32 | 0.07 | 0.89 | 0.009 |
| 2.5.3 Discontinue medications that may cause or exacerbate neuropathy. | 4.83 | 0.38 | 0.08 | 0.83 | 0.004 |
| 2.5.4 Avoid using medications that can cause kidney damage. | 4.89 | 0.32 | 0.07 | 0.89 | 0.009 |
| 2.5.5 For patients with diabetic nephropathy (DN), the choice of antihyperglycemic medications should be adjusted based on the estimated glomerular filtration rate (eGFR) levels. Sodium-glucose cotransporter 2 inhibitors (SGLT2i) are recommended. | 4.83 | 0.38 | 0.08 | 0.83 | 0.004 |
| 2.5.6 For patients with diabetic nephropathy (DN) to reduce urinary protein, angiotensin-converting enzyme inhibitors (ACEIs) or angiotensin II receptor blockers (ARBs) are the first-line medications. | 4.72 | 0.46 | 0.10 | 0.72 | 0.001 |
| 2.5.7 Imaging assessment of renal hemodynamic changes. | 4.83 | 0.38 | 0.08 | 0.83 | 0.004 |
| 2.5.8 If the patient's urinary albumin levels continue to rise and/or eGFR continues to decline and/or eGFR is less than 30 mL/min/1.73 m², referral to a nephrologist should be made. | 4.83 | 0.38 | 0.08 | 0.83 | 0.004 |
| **2.6 Urinary tract infection** | 4.94 | 0.24 | 0.05 | 0.94 | 0.047 |
| 2.6.1 Intensive blood glucose control, same as 2.1. | 4.78 | 0.43 | 0.09 | 0.78 | 0.015 |
| 2.6.2 Discontinue the use of sodium-glucose cotransporter 2 inhibitors (SGLT2i). | 4.61 | 0.50 | 0.11 | 0.61 | 0.006 |
| 2.6.3 Do not treat asymptomatic bacteriuria. | 4.44 | 0.78 | 0.18 | 0.61 | 0.002 |
| 2.6.4 Perform urine culture before antimicrobial treatment. | 4.72 | 0.46 | 0.10 | 0.72 | 0.009 |
| 2.6.5 Reduce bladder pressure, empty the bladder, manage vesicoureteral reflux, correct improper urination methods, and remove urinary stones. | 4.78 | 0.55 | 0.11 | 0.83 | 0.015 |
| **3.Health behavior intervention** | 4.89 | 0.32 | 0.07 | 0.89 | 0.312 |
| 3.1 Disease-related knowledge education | 4.78 | 0.43 | 0.09 | 0.78 | 0.050 |
| 3.1.1 Guide patients and their families to learn about diabetes, diabetic neurogenic bladder, and bladder management knowledge and skills. | 4.72 | 0.46 | 0.10 | 0.72 | 0.025 |
| 3.1.2 Educate patients and their families about the adverse outcomes of diabetic neurogenic bladder and the benefits of early bladder management | 4.72 | 0.46 | 0.10 | 0.72 | 0.025 |
| **3.2 Lifestyle intervention** | 4.83 | 0.38 | 0.08 | 0.83 | 0.088 |
| 3.2.1 Dietary management (1) Recommended management strategies from the Dietary Plan Execution Guidelines (2024 edition of the Chinese Guidelines for the Prevention and Treatment of Type 2 Diabetes). (2) Balance fluid intake and output. (3) Daily water intake of 1500-2000 ml, limit fluid intake 2-4 hours before bedtime. (4) Increase fiber-rich foods to avoid constipation. (5) Avoid bladder irritants such as tea, alcohol, coffee, and carbonated beverages. | 4.78 | 0.43 | 0.09 | 0.78 | 0.027 |
| 3.2.2 Exercise management (1) Low-intensity aerobic exercise (2) Low-impact resistance training (3) Core muscle training | 4.72 | 0.46 | 0.10 | 0.72 | 0.022 |
| 3.2.3 Sleep management (1) Maintain a regular sleep schedule. (2) Aim for 6-8 hours of sleep each night. (3) Create a conducive sleep environment (dark, quiet, with appropriate temperature and humidity). (4) Refer patients with significant sleep difficulties to a sleep specialist. | 4.61 | 0.50 | 0.11 | 0.61 | 0.017 |
| 3.2.4 Stress management  (1) Teach patients relaxation techniques and mindfulness meditation for self-relaxation. (2) Use sound therapy to regulate emotions by listening to pentatonic music after meals, five times a week for 30 minutes each session. (3) Refer patients with psychological issues to a mental health specialist for treatment. | 4.56 | 0.62 | 0.14 | 0.61 | 0.012 |
| 3.2.5 Avoid harmful substances and behaviors (1) Quit smoking (2) Abstain from alcohol; if drinking, avoid doing so on an empty stomach. (3) Avoid staying up late (4) Avoid wearing clothing that causes skin allergies, tight-fitting garments, and sitting baths. | 4.44 | 0.78 | 0.18 | 0.56 | 0.002 |
| 3.2.6 Interpersonal relationships: Establish good relationships with family, friends, patients, and the healthcare team to assist in disease management. | 4.50 | 0.79 | 0.17 | 0.61 | 0.007 |
| **3.3 Bladder training** | 4.89 | 0.32 | 0.07 | 0.89 | 0.113 |
| 3.3.1 Pelvic Floor Muscle Training (PFMT) | 4.78 | 0.55 | 0.11 | 0.83 | 0.063 |
| 3.3.2 Timed voiding (Indications: large capacity bladder, reduced sensation) | 4.72 | 0.57 | 0.12 | 0.78 | 0.037 |
| 3.3.3 Delayed voiding (Indications: overactive bladder) | 4.56 | 0.62 | 0.14 | 0.61 | 0.012 |
| **3.4 Self-monitoring** | 4.78 | 0.43 | 0.09 | 0.78 | 0.050 |
| 3.4.1 Bladder diary (1) Record drinking times, drinking amounts, urination times, urination amounts, and accompanying symptoms.  (2) Continuously record for 3 days. | 4.78 | 0.43 | 0.09 | 0.78 | 0.017 |
| 3.4.2 Self-monitoring of blood glucose: It can be scheduled for fasting, after three meals, before bedtime, or before and after vigorous exercise. | 4.83 | 0.38 | 0.08 | 0.83 | 0.028 |
| 3.4.3 Self-monitoring of urine: Seek medical attention promptly if experiencing discomfort or noticing abnormalities in urine color, consistency, or other characteristics. | 4.72 | 0.46 | 0.10 | 0.72 | 0.006 |
| **3.5 Social support** | 4.50 | 0.51 | 0.11 | 0.50 | 0.013 |
| 3.5.1 Encourage family and friends to provide emotional support to the patient. | 4.61 | 0.61 | 0.13 | 0.67 | 0.005 |
| 3.5.2 Achieve peer support through diabetes clubs and the use of the internet. | 4.56 | 0.62 | 0.14 | 0.61 | 0.004 |
| 3.5.3 Provide patients and their families with information related to public health service policies. | 4.39 | 0.70 | 0.16 | 0.56 | 0.002 |
| 3.5.4 Provide patients and their families with information related to medical insurance. | 4.33 | 0.69 | 0.16 | 0.44 | 0.001 |
| 1. **Follow-up and evaluation** | 4.33 | 0.77 | 0.18 | 0.56 | 0.062 |
| **4.1General follow-up content and frequency** | 4.67 | 0.49 | 0.10 | 0.67 | 0.007 |
| 4.1.1 Monitor BMI, waist circumference, blood pressure, and blood glucose monthly. | 4.39 | 0.78 | 0.18 | 0.56 | 0.001 |
| 4.1.2 Test HbA1c every three months until the target is reached, and every six months thereafter. | 4.50 | 0.71 | 0.16 | 0.61 | 0.002 |
| 4.1.3 Monitor blood lipids once a year. | 4.56 | 0.62 | 0.14 | 0.61 | 0.004 |
| **4.2 Specialized follow-up content and frequency** | 4.83 | 0.38 | 0.08 | 0.83 | 0.035 |
| 4.2.1 Urinalysis once every 2 months. | 4.50 | 0.71 | 0.16 | 0.61 | 0.002 |
| 4.2.2 Urinary system ultrasound and residual urine volume measurement once every 6 months. | 4.72 | 0.46 | 0.10 | 0.72 | 0.015 |
| 4.2.3 Kidney function and urodynamic tests once a year. | 4.61 | 0.61 | 0.13 | 0.67 | 0.011 |
| 4.2.4 It is recommended to use imaging urodynamic testing; if conditions do not permit, asynchronous bladder-urethra imaging combined with urodynamic testing should also be performed. | 4.56 | 0.62 | 0.14 | 0.61 | 0.007 |
| **4.3 Evaluation indicators** | 4.78 | 0.43 | 0.09 | 0.78 | 0.021 |
| 4.3.1 Primary outcome indicators: bladder residual urine volume, Neurogenic Bladder Symptom Score (NBSS) | 4.72 | 0.46 | 0.10 | 0.72 | 0.010 |
| 4.3.2 Secondary outcome indicators: metabolic control indicators (glycated hemoglobin, blood pressure, blood lipids), Diabetes Self-Management Questionnaire (DSMQ) scores, quality of life (SF-Qualiveen scale). | 4.72 | 0.46 | 0.10 | 0.72 | 0.010 |

Abbreviations：SD, standard deviation; CV, coefficient of variation.
